# Anti‐fungal bandages containing cinnamon extract

**DOI:** 10.1111/iwj.13090

**Published:** 2019-02-15

**Authors:** Jubair Ahmed, Esra Altun, Mehmet O. Aydogdu, Oguzhan Gunduz, Laxmi Kerai, Guogang Ren, Mohan Edirisinghe

**Affiliations:** ^1^ Department of Mechanical Engineering University College London London UK; ^2^ Department of Metallurgical and Materials Engineering University of Marmara Istanbul Turkey; ^3^ School of Life and Medical Sciences University of Hertfordshire Hertfordshire UK; ^4^ School of Engineering and Technology University of Hertfordshire Hertfordshire UK

**Keywords:** anti‐fungal, bandages, Cinnamon, healthcare, mass production

## Abstract

Cinnamon‐containing polycaprolactone (PCL) bandages were produced by pressurised gyration and their anti‐fungal activities against *Candida albicans* were investigated. It was found that by preparing and spinning polymer solutions of cinnamon with PCL, fibres capable of inhibiting fungal growth could be produced, as observed in disk diffusion tests for anti‐fungal susceptibility. Fascinatingly, compared with raw cinnamon powder, the novel cinnamon‐loaded fibres had outstanding long‐term activity. The results presented here are very promising and may indeed accelerate a new era of using completely natural materials in biomedical applications, especially in wound healing.

## 1 INTRODUCTION

Even before the discovery of modern antibiotics, anti‐microbial agents, for example, spices, have existed in consumable form. Several commonly eaten foods have the uncultivated ability to inhibit microbial growth, which remained unknown to the consumer. These spices are not only abundant but also come at relatively little cost whilst being completely natural and having a minor impact on the environment. Cinnamon (*Cinnamomum*) is a spice acquired from the inner dried bark of several species of trees indigenous to South Asia and China and belongs to the genus *Cinnamomum*.^1^ Cinnamon is grown commercially and is used in cosmetics, as a flavouring additive in a wide variety of cuisines worldwide, and as an aromatic condiment. The history of cinnamon usage dates back thousands of years where references to it are found in the Bible. The ancient Egyptians even used the spice as embalming fluid for the wealthy deceased.^1^ Arguably, two most prevalent types of cinnamon exist, *Cinnamomum verum* (Ceylon) *and Cinnamomum cassia*. Ceylon cinnamon is considered amongst most to be the “true cinnamon” whilst *cassia* cinnamon is most commonly found in international commerce and thus holds a lower trading value.^2^ The principal chemical component of cinnamon, cinnamaldehyde, gives the spice its unique aroma and flavour; *cassia* variants contain more cinnamaldehyde in their essential oil.^3^ Historically, cinnamon has been long used as a traditional medicine in the treatment of many conditions, such as bronchitis, rheumatism, and neuralgia.^4^ Furthermore, many modern scientific articles report on the ability of cinnamon to be antidiabetic, antioxidant, anti‐inflammatory, anti‐fungal, anti‐HIV, improve heart function, pro‐wound healing, and anti‐cancer.^5^, ^6^, ^7^, ^8^, ^9^, ^10^ It has been reported that cinnamaldehyde holds remarkable anti‐microbial activity.^11^, ^12^, ^13^, ^14^

*Candida albicans* is found in abundance in the human gut flora of around 50% of all healthy adults and is an opportunistic pathogenic yeast.^15^, ^16^ *C. albicans* is known to cause human infections such as candidiasis, which occurs as a result of its overgrowth and is a contributing factor in tooth decay.^17^, ^18^ Furthermore, *C. albicans* is the most common fungal species found to form as a biofilm on medical implants and is the cause of many implant failures.^19^ Cost of treatment for patients only increases because of the rise of hospital‐acquired infections.^20^ The usage of bandages and gauzes still remains a highly prevalent foundation of wound care in many countries.^21^, ^22^ The anti‐fungal effect of cinnamon‐blended polymeric fibres on *C. albicans* is studied in this article. There is an abundance of work completed in anti‐microbial materials, but here we investigate a completely natural solution.^23^, ^24^

## 2 MATERIALS AND METHODS

### 2.1 Fibre preparation

**Figure 1:**
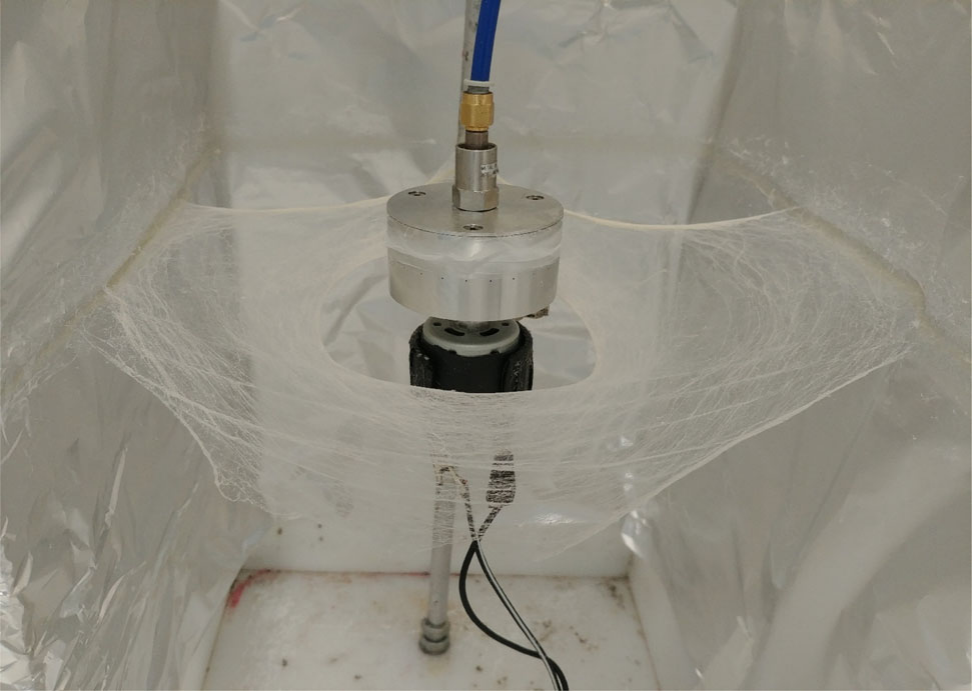
Picture of pressurised gyration setup, showing a high yield of polycaprolactone fibres resembling bandages, produced with 4 mL of polymer solution

Solvent extraction via chloroform (CAS Number: 67–66‐3, Sigma Aldrich, UK) was carried out in order to formulate varying concentrations of cinnamon in solution. Three cinnamon (cassia) extractions (C1, C2, and C3) of differing concentrations where prepared from ground cinnamon powder (JustIngredients, UK). The extraction process involved mixing 6, 9, and 12 g into 24 mL of chloroform; the solution was vortexed, excess cinnamon that was not dissolved into the solvent was separated by addition of 10 mL distilled water and centrifugation at 2000 rpm for 15 minutes. Four concentrations of fibres were tested in this investigation: virgin PCL fibres with no cinnamon, C1, C2, and C3 fibres with 6, 9, and 12 g cinnamon per 24 mL of chloroform, respectively. PCL (Mn 80 000, Sigma Aldrich, UK) was added to the cinnamon extract solutions to make a concentration of 15% (w/v) and magnetically stirred overnight to produce a polymer solution. The solutions were spun with a laboratory‐pressurised gyration set up that consists of a small (60 × 35 mm^2^) aluminium cylindrical vessel with multiple, narrow (0.5 mm) perforations that is connected to a gas inlet and a high‐speed motor to produce fibre meshes.^25^ A 4 mL volume of the polymer solution was spun with pressurised gyration for 20 seconds in order to obtain a fibrous square bandage under ambient conditions (24°C and 42% relative humidity). Figure 1 depicts the fibre‐forming setup that has completed a single spin of 4 mL PCL polymer solution.

### 2.2 Anti‐fungal testing

A total of 100 mg of cinnamon‐containing fibres (C1, C2, and C3 samples) were tested at different concentrations in 10 mL of Mueller‐Hinton agar (Sigma Aldrich, UK). A volume of 250 μL of *C. albicans* (population 4‐5 × 10^6^) was distributed within the standard Petri dish. The fibres were placed in the centre of the Petri dish in order to determine any inhibition zones between the fungus and the samples. The agar plates were incubated at 37°C. As a negative control, the fibres containing PCL only were also used and inhibition zones analysed. As a positive control, raw material of cinnamon in powder form was tested as described previously. Images of the agar plates were taken following 48 hours from incubation, inhibition zones were calculated as areas using Image J software. Scanning electron microscopy (Hitachi S‐3400n, Tokyo, Japan) was used to analyse the morphology of the fibres. Unless otherwise labelled, an accelerating voltage of 5 kV was used to image the samples which were gold sputter coated for 180s (Q150R ES Quorum Technologies Ltd., Laughton, England).

## 3 RESULTS

The anti‐fungal capability of cinnamon was tested against *C. albicans* where inhibition areas were used to determine the effectiveness of each anti‐fungal substance. Larger inhibition zones were indicative of a greater ability for the material to kill the fungal population, these have been compared in the following results.

Inhibition zones for each of the samples were compared for their effectiveness in preventing fungal growth. Comparisons were made on the average inhibition zone that was calculated as an area for fair comparison. Larger inhibition zones showed greater anti‐fungal efficacy.

Fungus, like all other microbes, comes into contact with the surface of materials. It is, therefore, important to study the fibres to identify if any differences in fibre morphology could have had an effect on the anti‐fungal activity.

Inhibition zones clearly show anti‐microbial effect, but this could be because of a number of factors other than the cinnamon extract. To eliminate doubt of the anti‐fungal ingredient, virgin PCL fibres containing no cinnamon extract were seen under microscopy and compared with cinnamon‐extracted PCL fibres.

How long the anti‐fungal effect lasts is also an important consideration. Following 504 hours of initial incubation, the samples were rephotographed to observe the longevity of the anti‐fungal influence.

## 4 DISCUSSION

**Figure 2:**
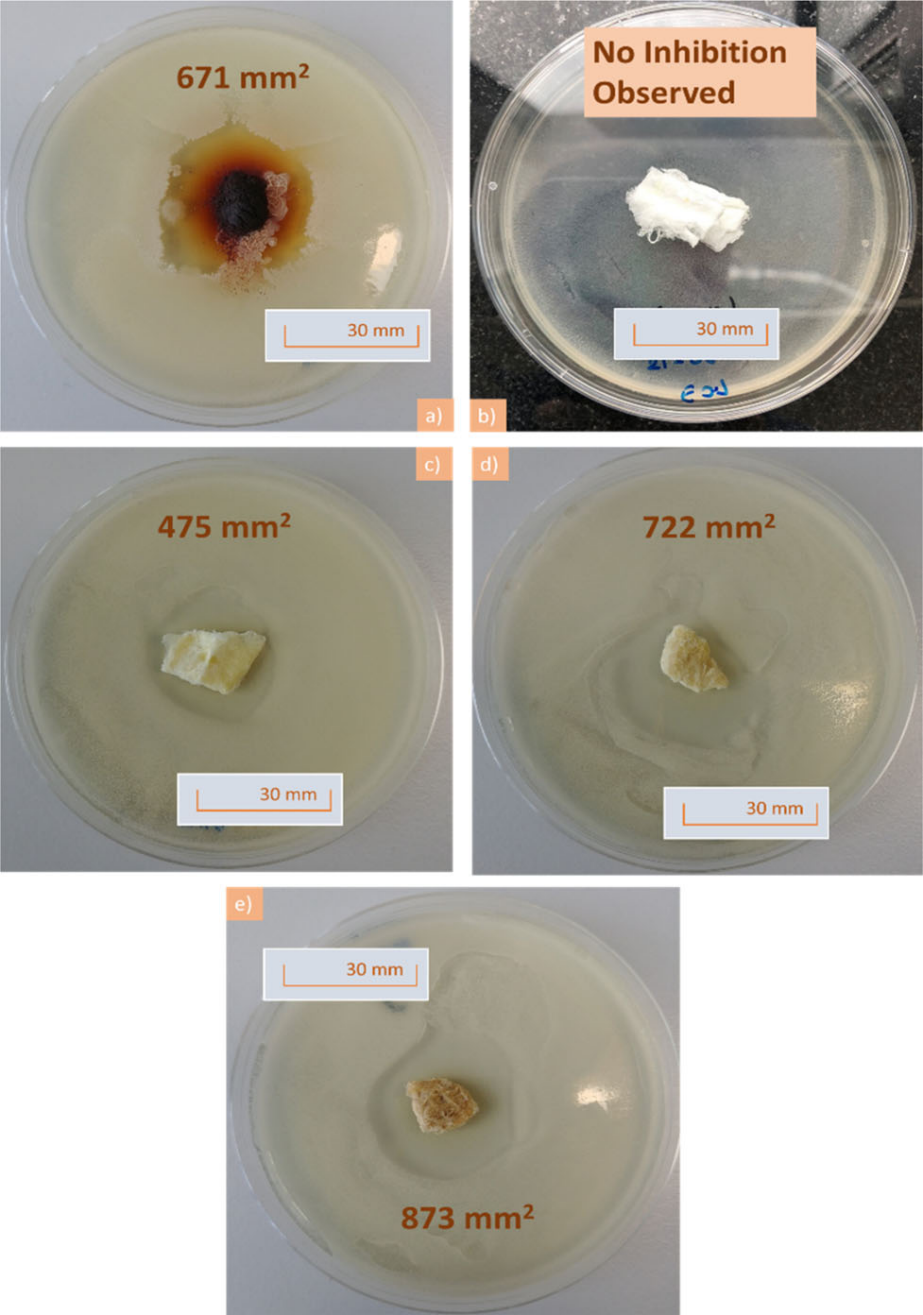
Pictures following 48 hours of incubation showing plates containing; A, ground cinnamon powder, B, virgin polycaprolactone fibres, showing lack of an inhibition area, C, C1 cinnamon‐extracted fibres, D, C2 cinnamon‐extracted fibres, E, C3 cinnamon‐extracted fibres and in each case, 100 mg of sample was investigated. For each plate, the inhibition area value is given

All of the samples cinnamon were anti‐fungal as evident from Figure 2 as it all shows inhibition areas devoid of fungal growth. Figure 2A shows cinnamon powder demonstrating that, as expected, the raw ingredient does in fact have an anti‐fungal effect. Virgin PCL fibres without any cinnamon extract are shown in Figure 2B and there is no zone of inhibition as PCL itself is not anti‐microbial, furthermore, it validates that the chloroform from the fibre‐manufacturing process has completely evaporated as otherwise this would have led to an inhibition zone. It can also be seen that larger inhibition zones surround the cinnamon‐loaded fibres (Figure 2C‐E) as the concentration in the fibres increases from 6 to 12 g/24 mL. As expected, using more cinnamon powder in the extraction process leads to a higher concentration of cinnamaldehyde within the solution and subsequently this is incorporated into the fibre bandages.

**Figure 3:**
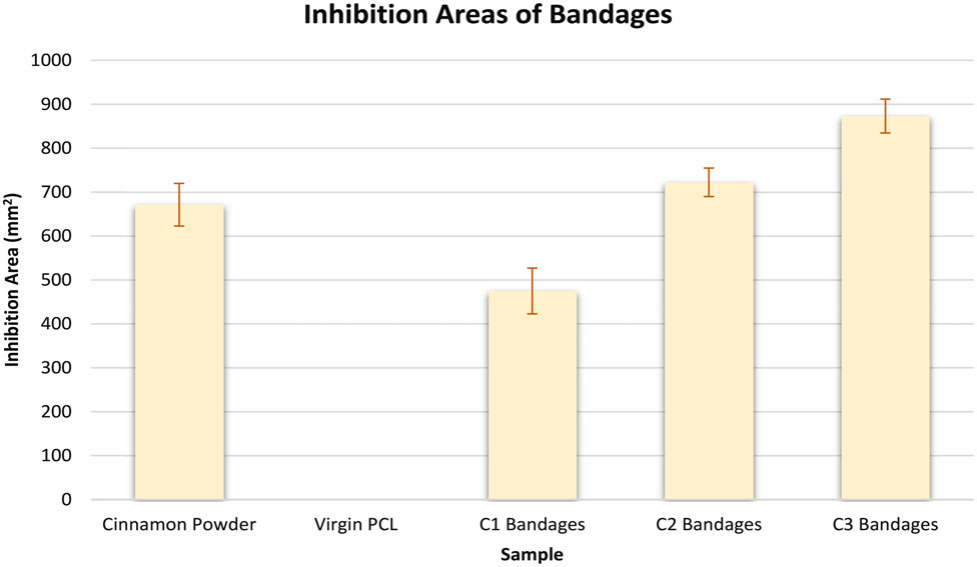
Graph comparing inhibition areas of the different samples, shown in Figure 2, n = 3

Virgin PCL fibres had an absence of any inhibition zones as expected from the negative control as these bandages were free from cinnamon extract (Figure 3). Raw cinnamon powder expresses a larger inhibition area of 671 ± 49 mm^2^ and compared with the C1 cinnamon‐loaded fibres (475 ± 52 mm^2^). However, as the concentration within the fibres increases from 6 to 12 g/24 mL, there is enhanced potency in the anti‐fungal activity as confirmed by larger inhibition areas (722 ± 32 and 873 ± 39 mm^2^, respectively). Extracts of cinnamon contain several active compounds including cinnamaldehyde and tannins.^26^ Tannins are known to inhibit growth of certain bacterial strains such as *Staphylococcus aureus*.^27^ Cinnamaldehyde too is anti‐microbial because of its activity in inhibiting cell wall synthesis of microbes.^28^ During the extraction process, the powder and cellulose of the cinnamon were discarded leaving only the chemical extract. The results here, therefore, suggest that the anti‐fungal effect may indeed be of chemical origin. Having a lower minimum non‐cytotoxic concentration than many other natural anti‐microbial materials, it is unlikely that the anti‐fungal effect from cinnamon is because of a toxic effect.^29^

**Figure 4:**
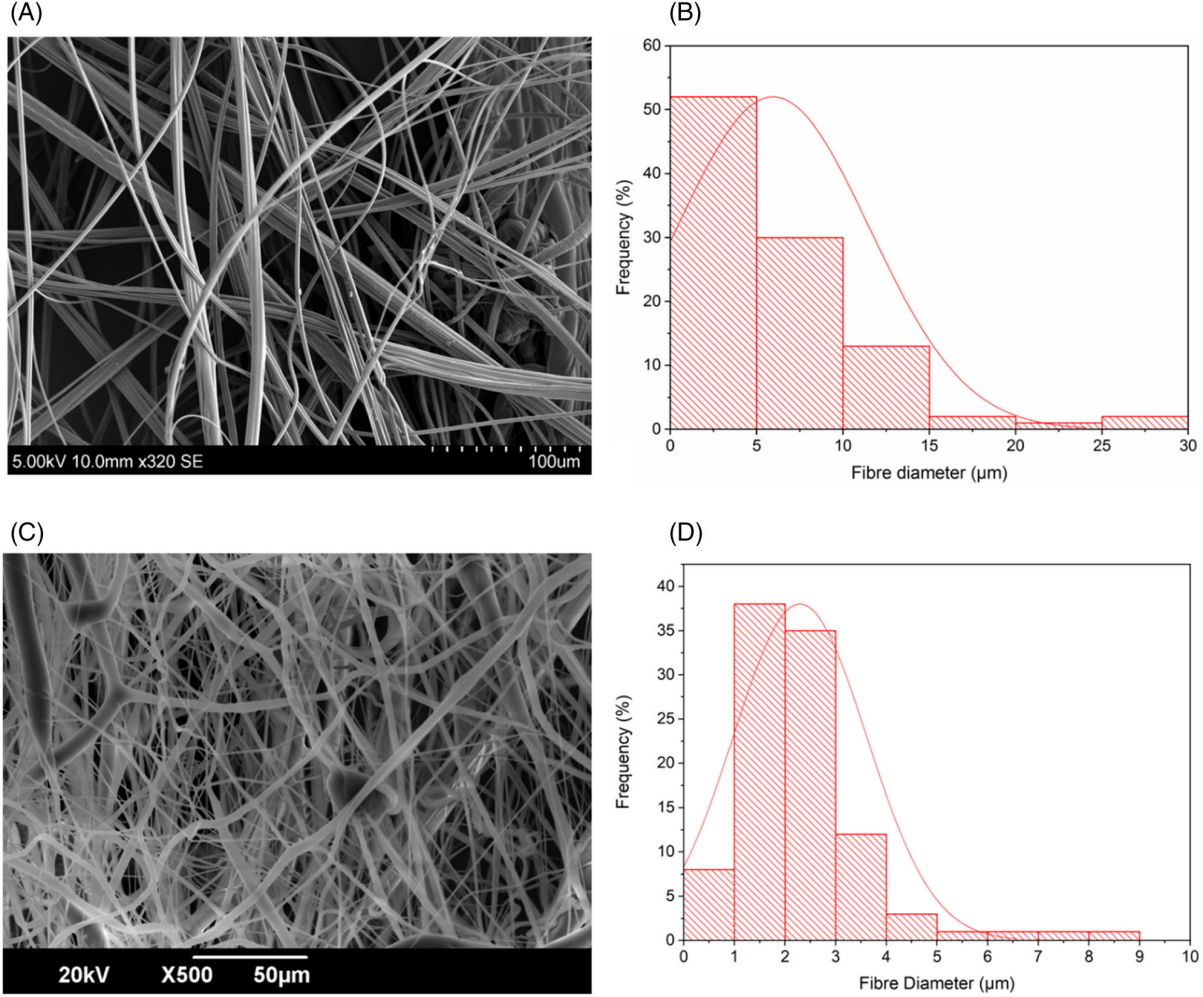
Scanning electron microscopy images of pressure spun fibres at 0.1 MPa: A, virgin polycaprolactone (PCL) fibres, B, diameter distribution of the virgin PCL fibres, C, PCL fibres with cinnamon‐extract (C2), and D, diameter distribution of the cinnamon‐loaded PCL fibres, in all cases, n = 100

The average fibre diameter of the virgin PCL fibres is 6.0 ± 2.6 μm, the diameter distribution of these fibres is shown in Figure 4B. These thin fibres are able to carry the active ingredients of cinnamon whilst acting as a physical barrier to microbes such as fungi. These fibres contain nano‐pores that are the result of rapid solvent evaporation and subsequent micro‐droplet formation on the fibre surface as detected in our previous work.^30^ These pores increase the available surface area to volume ratio of the bandages and also allow for the free movement of exudate in and out of the bandage, which is a highly desired characteristic for a wound dressing.^31^, ^32^ Figure 4B shows cinnamon‐extract loaded PCL fibres (9 g), which have an average fibre diameter of 2.3 ± 1.3 μm. Compared with the virgin PCL fibres, the cinnamon‐loaded fibres are thinner and have a smaller diameter distribution (Figure 4D) meaning that the fibre thicknesses are more uniform. Lower‐diameter fibres can be more beneficial in releasing active ingredients because of the higher surface area to volume ratio they afford.^33^

**Figure 5:**
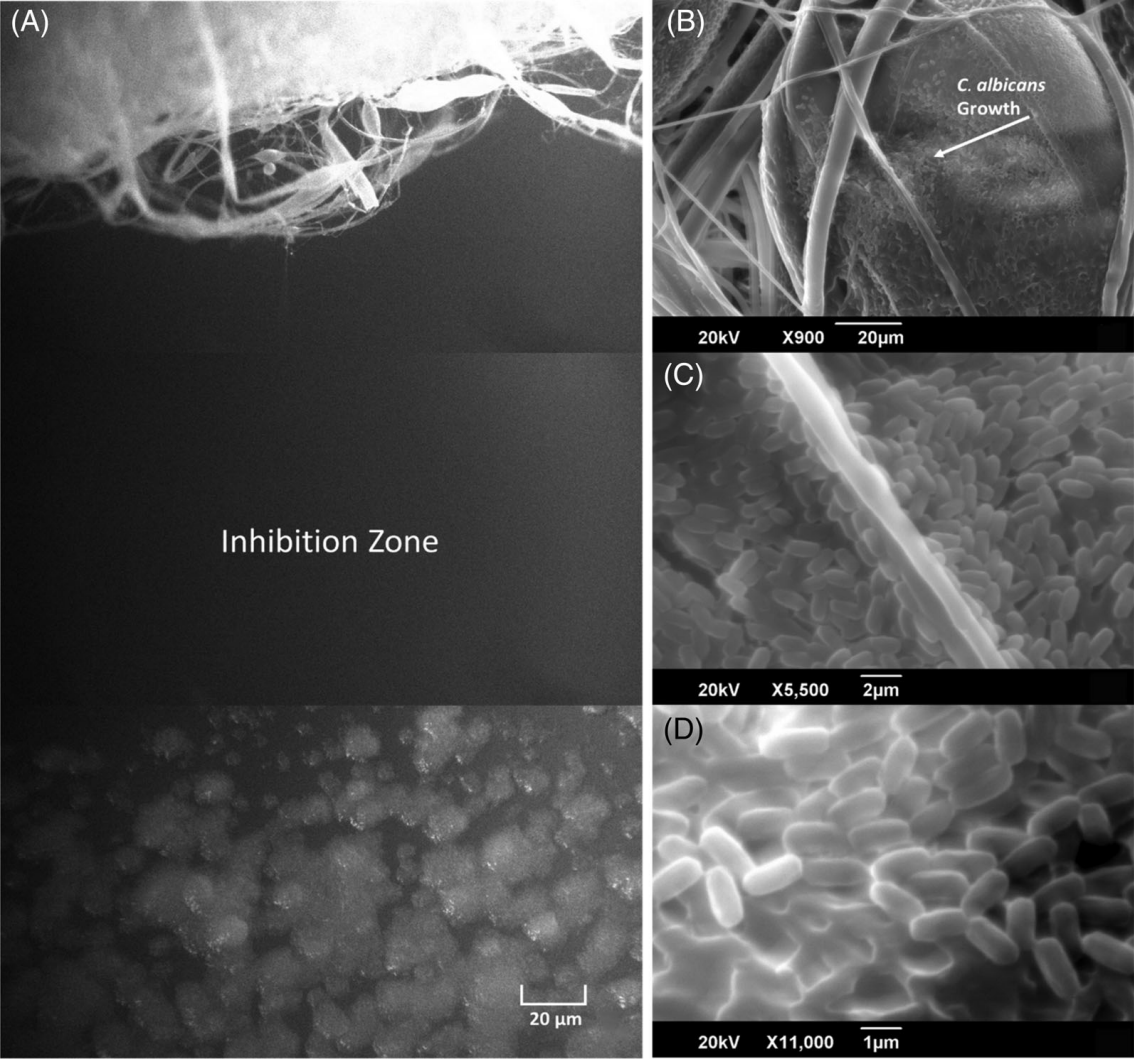
Fluorescence microscopy video stills of: A, C1 extracted cinnamon fibre showing a clear inhibition zone with no fungal growth, scanning electron microscopy images of: B, C, candida growth over virgin PCL fibres, arrow marked in B, and D) high magnification image showing close‐up of *Candida. albicans* cell growth on virgin PCL fibre surface

It is evident from Figure 5 that there is a clear inhibition zone in between the extracted cinnamon‐containing fibres where *C. albicans* cells have failed to survive. The images consist of three frames taken from a video clip (available in Supporting Information), which pans an area between the fibre and the fungi. Clearly, the extracted cinnamon‐containing fibres are successful in denying fungal growth here.

**Figure 6:**
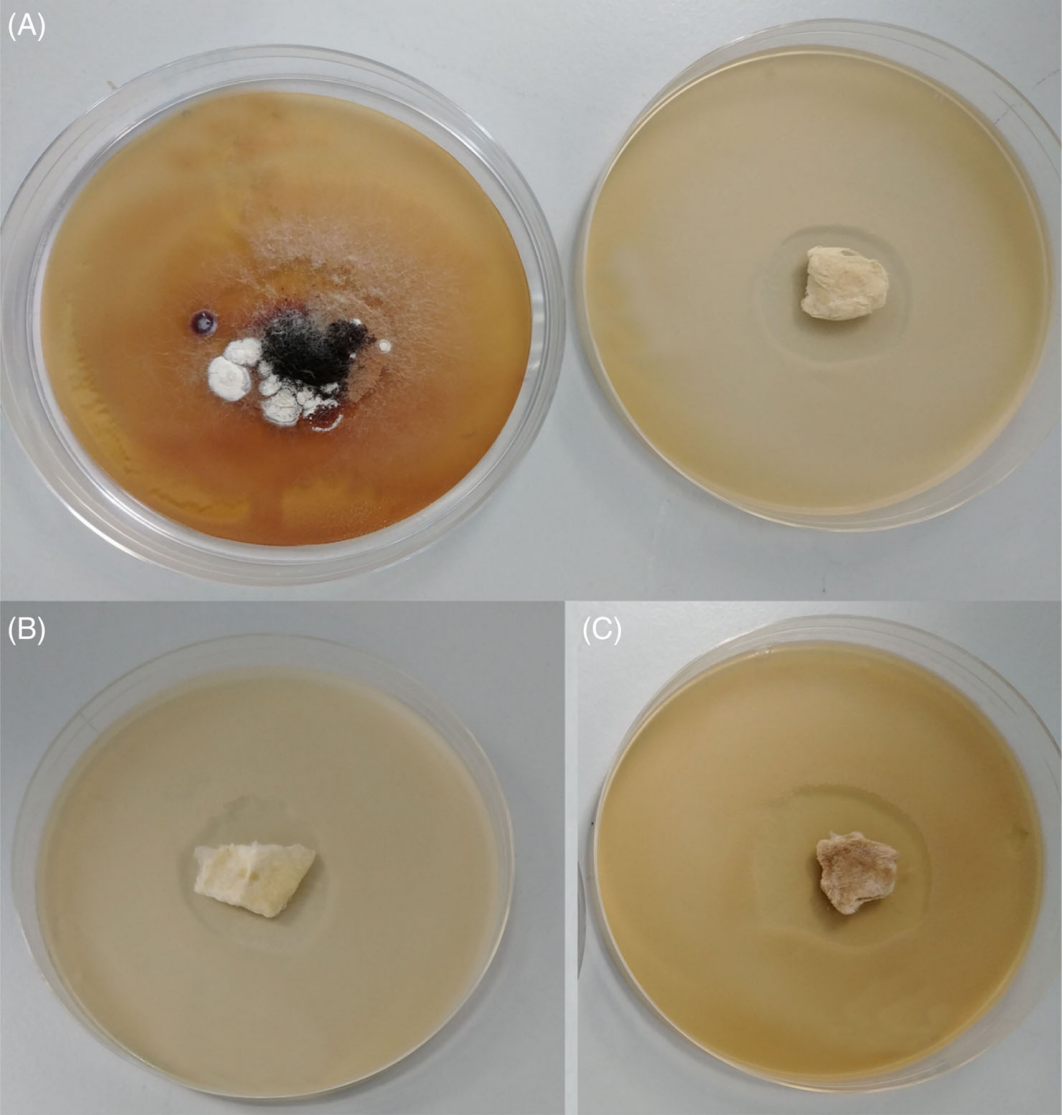
Images taken 504 hours after initial incubation comparing: A, C1 extracted cinnamon‐containing fibres (right‐hand side) with raw cinnamon powder (left‐hand side) B, C2 extracted cinnamon‐containing fibres C, C3 extracted cinnamon‐containing fibres. All Petri dishes shown are 90 mm in diameter

Very interestingly, following 3 weeks of incubation, the cinnamon powder solutions show a regrowth of fungal colonies indicating that the anti‐fungal effect of ground cinnamon powder is only temporary and given time, fungi are able to grow into the previously formed inhibition zones (Figure 6). All the extracted cinnamon fibres (6, 9 and 12 g) still retain the exactly the same inhibition zones, demonstrating that the anti‐fungal effect is not short‐term and even after 3 weeks of 37°C incubation, there is no regrowth of fungal communities. One possible explanation for the regrowth of fungi in the cinnamon powder is that the fungi are initially repelled from the powder, but the surviving fungi are able to grow in numbers and invade the powder that acts as their food source for further growth. In any case, we have successfully demonstrated here that we can obtain lasting anti‐fungal activity with cinnamon‐loaded fibres. Compared with raw cinnamon, there is no regrowth of fungi following 3 weeks.

## 5 CONCLUSIONS

From the results presented in this work, it is obvious that the gyrospun cinnamon‐extract‐containing bandages have a strong anti‐fungal effect. Compared with the negative control (Virgin PCL), there is a far greater inhibition area associated with these fibres. Cinnamon‐loaded fibres had strong anti‐fungal activity that lasted in excess of 3 weeks, something that could not have been achieved if not in the fibre form. This work opens up the possibility for crude unpurified extracts of natural materials to be used in wound care and other anti‐microbial filtering applications. The use of such natural materials alleviates fear and concern of long‐term consequences and side effects as these materials have been used by humans for many millennia. Spinning them into bandage‐like mats shows the manufacturing and health care capability of such materials is very effective (as shown here) when incorporated into a fibrous patch, environmentally unfriendly purification can be avoided. Our work in progress embraces other natural medicinal materials such as garlic, cardamom, and aniseed.

## CONFLICT OF INTEREST

All the authors agree in that there is no conflict of interest to declare.

## Supporting information


**APPENDIXS1**: Video
